# Fresh Start: A pilot, randomized controlled trial of a virtual reality (VR) experience to develop discrepancy and elicit change talk in brief motivational interventions for hazardous drinking among young adults

**DOI:** 10.21203/rs.3.rs-10540866/v1

**Published:** 2026-08-03

**Authors:** Tyler Blake Wray, Amy C. Traylor, Molly Magill, Nancy Barnett, Christopher Kahler, Ruben Martinez

**Affiliations:** Brown University; University of Alabama System; Brown University; Brown University; Brown University; Brown University

**Keywords:** Virtual reality, motivational interviewing, brief intervention, alcohol use, hazardous drinking, young adults, discrepancy, change talk

## Abstract

**Background:**

Alcohol use is the leading risk factor for premature death among young adults globally. Brief motivational interventions (BMIs) inspired by Motivational Interviewing (MI) are effective but produce modest and short-lived effects, prompting interest in ways of enhancing the effects of these interventions. Developing self-discrepancy, the perception that one’s current behavior conflicts with important values or life goals, is a key mechanism in MI. Virtual Reality (VR) has the potential to help elicit self-discrepancy by immersing users in vivid, interactive experiences that encourage deep reflection. We designed Fresh Start, a VR experience that could be used in MI sessions to help develop discrepancy and elicit change talk. This paper describes the protocol for a pilot randomized controlled trial to evaluate the tolerability, acceptability, and preliminary efficacy of Fresh Start when integrated into BMIs on hazardous drinking among young adults.

**Methods:**

Hazardous drinking young adults (N=120; ages 18–34) referred to the University of Alabama’s Collegiate Recovery and Intervention Services will be randomized 1:1 to: (1) BMI with VR (Fresh Start) or (2) time-matched BMI only (control). Tolerability, acceptability, and feasibility of the VR experience will be assessed in a post-session survey. Alcohol use and problems will be assessed at 1, 2, 3, and 6 months post-intervention.

**Discussion:**

This study will produce one of the first rigorous evaluations of a VR experience designed to help build motivation to change drinking by eliciting self-discrepancy. Results will provide important data on tolerability and acceptability outcomes of VR-enhanced BMI, as well as its preliminary efficacy, which are necessary for planning a larger effectiveness trial.

## Administrative information

**Table:** 

**Title {1a}**	Fresh Start: A pilot, randomized controlled trial of a virtual reality (VR) experience to develop discrepancy and elicit change talk in brief motivational interventions for hazardous drinking among young adults
**Structured summary {1b}**	See Abstract
**Protocol version {2}**	Version 1, May 2026
**Roles and responsibilities {3a}**	TBW, AT, NPB, MM, CK, and RM serve as the core scientific team for this trial. TBW will provide overall leadership of the project. AT provides expertise on VR- assisted treatments for addiction. NPB contributes expertise in brief alcohol interventions and will assist in training and supervising study counselors. MM contributes expertise in mechanisms of behavior change and will assist in fidelity coding of selected sessions. CK will assist with data analysis and contributes expertise in integrating BMI with additional intervention components. RM provides expertise in implementation science methods.
**Trial sponsor {3b}**	National Institute on Alcohol Abuse and Alcoholism (NIAAA), https://www.niaaa.nih.gov/
**Role of sponsor {3c}**	Study sponsors played no role in the study design, the collection, management, analysis, or interpretation of data, writing of the report, or the decision to submit the report for publication.
**Coordinating site and oversight {3d}**	Dr. Wray serves as overall PI and leads all research activities from Brown University, where all data collection and participant management systems are housed. Dr. Traylor serves as site PI at the University of Alabama's Collegiate Recovery and Intervention Services center, where intervention delivery and participant enrollment will occur. Dr. Barnett serves as primary clinical supervisor for intervention delivery. The full study team (Wray, Traylor, Barnett, Magill, Kahler, and Martinez) will jointly oversee all scientific decisions through quarterly meetings. No independent data monitoring committee is in place. See item {28a} for rationale.

## Open science

### Trial registration {4}

[Record is in review, but not yet released by CT.gov. Will be added prior to publication.]

### Protocol and statistical analysis plan {5}

The trial protocol and statistical analysis plan will be available both through ClinicalTrials.gov and this manuscript, which will be available via the journal’s open access platform upon publication.

### Data sharing {6}

After all participants have completed study procedures, a de-identified archival dataset will be created and uploaded to the NIAAA Data Archive in accordance with institute policy. This dataset will include only those participants who opted into allowing their data to be archived at enrollment. The dataset will be shared upon request after an embargo period of 2 years. Analysis scripts associated with all publications will also be made available upon request.

### Funding and conflicts of interest {7a}

This work was supported by NIAAA grant R01AA031282 to TBW. The sponsor has no role in the design of the study, data collection, analysis, or interpretation, or in the writing of this and other manuscripts.

### Conflicts of interest {7b}

TBW is Founder and President of Real Recovery VR, Inc., a company that sells a VR platform for counselors to assist in treating patients with addiction. MM is a member of the Scientific and Clinical Advisory Board for Real Recovery VR, Inc.

### Dissemination policy {8}

Results of the primary outcomes identified in this trial will be published in relevant scholarly journals. Investigators will also present results at relevant national conferences (e.g., Research Society on Alcoholism, Society for Behavioral Medicine). Results will be uploaded into the trial registry after they are available. A final, de-identified archival dataset will be uploaded to the NIAAA Data Archive and made available to requesting investigators after an embargo period of 2 years.

## Introduction

### Background and rationale {9a}

#### Despite declines, many young adults drink at hazardous levels and experience alcohol-related consequences

Alcohol use is the leading risk factor for premature death among young adults globally. [1] In the United States (US) from 2006–2010, alcohol use directly caused over 16,000 deaths in those ages 18–34 [2] and is estimated to result in over 500,000 injuries each year.[3] Heavy drinking is also widespread in this age group, with 37% reporting at least one binge drinking episode (>5 drinks for men or >4 drinks for women on a single occasion) in the past month.[4] Research exploring ways to reduce hazardous drinking among young adults continues to be a top public health priority.

#### Brief interventions based in motivational interviewing reduce hazardous drinking

Meta-analyses have shown that MI-inspired interventions produce meaningful reductions in alcohol use and problems compared to no-treatment or placebo controls (*d=*0.25–0.53), across populations such as college students, emergency room patients, and individuals in treatment for substance use disorder.[5, 6] MI-inspired techniques are also among the most frequently used among substance use clinicians in community settings,[7] likely a result of past and ongoing dissemination efforts.[8, 9] These techniques form the foundation of brief motivational interventions (BMIs),[10–12] which are effective in helping a variety of populations, including young adults, reduce their alcohol use across a number of settings.[6, 13–15] However, MI’s effects tend to be roughly equivalent when compared to more active treatments. [5, 6] More recent meta-analyses using individual participant data have further suggested that BMI effects on drinking may be smaller and shorter-lived than earlier estimates indicated.[16, 17] This combination of high utilization among counselors with somewhat limited effects has prompted interest in ways to enhance the effects of BMIs.[18, 19] Together, this research suggests that MI-based interventions are effective and popular, but that adjunctive techniques may be needed to produce more significant, longer-term changes in alcohol use.

#### Developing discrepancy is a powerful technique in MI interventions

In MI-inspired interventions, counselors help patients explore and resolve their ambivalence about their drinking by taking a collaborative approach, expressing empathy, and listening reflectively, as well as by applying specific techniques, such as asking open-ended questions that encourage reflection on particular topics (e.g., values, important life goals).[20] Although it is still unclear exactly how MI helps clients reduce their drinking,[21] one potentially important factor is the degree of discrepancy clients feel between their behavior now and their broader values and goals.[22] Discrepancy refers to a “deepened, conscious incongruity between one’s actual and ideal drinking patterns” (p. 80).[22] Developing this sense of discrepancy is a key goal of MI. [23] A meta-analysis of MI mechanisms showed that, aside from client language about making a change (i.e., change talk), patients’ post-session report of discrepancy was one of the only patient-level factors associated with better outcomes in MI.[24] More recent studies also support these findings.[25, 26] For instance, McNally et al. [22] showed that actual-ideal discrepancy, or patients’ perception that their current behavior does not match their ideal behavior, was one of two types of discrepancy associated with greater reductions in drinking after a BMI.

Several MI techniques are used to develop discrepancy. Personalized, normative feedback helps highlight the inconsistency between clients’ own drinking and perceived norms of others’ drinking (self-other discrepancy),[27] and some evidence suggests that BMIs that incorporate feedback show greater reductions in drinking than those that do not.[13, 28–31] Another way to develop a deep and meaningful sense of discrepancy involves asking evocative, open-ended questions that help clients consider the ways in which their current behavior aligns or conflicts with important personal values or goals. [18,40] “Looking forward,” for example, involves helping clients imagine what they would like life to look like in the future and to reflect on whether their current behavior gets them closer to or further away from those goals. These MI strategies can be powerful tools that help clients reflect and provide clarity about their priorities and the consequences of their decisions.[23, 32] However, these discussions are not always included in BMIs, [30, 33] and are only as powerful as clients’ engagement, an important consideration given that such interventions often target those who are ambivalent about change.

#### Virtual reality (VR) could enhance MI by providing a transformative experience that helps develop a strong sense of discrepancy

VR refers to a computer-generated experience that provides an extensive, surrounding, and vivid illusion of reality to human users.[34] It gives users the feeling of being immersed in a new environment by realistically mimicking as many of their senses as possible (e.g., stereoscopic vision, head movement/tracking, stereo sound).[35] Integrating these elements gives users the feeling of presence, or a convincing sense that the virtual environment is real.[36] The sense of presence naturally arises from this particular blend of digital elements and is not dependent on users’ belief that it is or will feel real. [37] [38]

VR has been used in the treatment of a variety of mental health conditions, including post-traumatic stress disorder, specific phobias, social phobia, panic disorder, depression, and complicated grief.[39–42] Currently, the most well-supported use of VR is for the delivery of exposure therapy for anxiety disorders. In this model, VR-enhancements are used to immerse patients in feared situations, allowing patients to gradually confront them and acquire new safety learning that contradicts their anxious beliefs about the dangerousness and intolerability of those situations.[43–45] Importantly, VR can help facilitate exposures for many feared situations or objects that are difficult to achieve through in vivo exposures (e.g., flying, public speaking).[46] Meta-analyses have shown that these VR-assisted exposures are as effective as in vivo exposure [47–49] and generalize to real life.[39, 50]

VR experiences have been developed to help treat addictions [51–54], with experiences designed to facilitate various therapeutic goals, including helping patients identify triggers or practice coping strategies,[55] learn and practice mindfulness skills,[56] or to facilitate cue exposure.[57, 58] Some have shown promising results, but evidence of efficacy is almost exclusively from very small pilot trials with limited follow-up.[52, 59, 60]

Beyond mere exposures, VR could also be used to create more transformational and memorable experiences that help motivate patients to change.[61, 62] For example, it has been used to create experiences aimed at shifting users’ perspectives, such as experiences designed to inspire awe and wonder that have been shown to encourage pro-social behaviors.[63–65] Other VR experiences have been developed to help change users’ attitudes and create empathy by adopting another’s perspective. [42, 66–68] These findings illustrate the potential VR has for facilitating transformational and memorable experiences capable of creating positive motivation and behavior change.[61, 69–71] Designing a VR experience that helps users reflect on their drinking and its consequences could be a potent tool for helping develop discrepancy during MI-inspired treatments.

#### Fresh Start: A VR experience to develop discrepancy and elicit change talk in young adults

Given VR’s potential, we designed *Fresh Start*, an immersive experience for use in BMI sessions to help elicit change talk among young adults by developing discrepancy. Our vision was that counselors could use this tool as one strategy (among others) to help clients explore and resolve their ambivalence about their alcohol use by illustrating the conflict between important life goals and participants’ current behavior. To achieve this, we worked to ensure the experience (1) was consistent with the “spirit” of MI (i.e., be collaborative, emphasize autonomy, evoke the user’s priorities, convey empathy) [20], that it (2) inspired exploration and curiosity, and that it (3) encouraged users to meaningfully grapple with the tension between their drinking and important life priorities. Fresh Start is an immersive VR experience in which users select objects representing personal life priorities and then must load them into a moving truck that is already partially occupied by a physical representation of their own alcohol use. This premise forces users to physically resolve the trade-off between drinking and what matters most to them, making discrepancy concrete in a way that discussion alone may not.

### Objectives {10}

The objectives of this trial are to:

- Assess the tolerability, feasibility, and acceptability of the Fresh Start VR experience when used in BMI sessions among college students who have been mandated by their university to receive alcohol screening and brief intervention. These constructs will be assessed through refusal rates and post-session surveys.
- Test whether BMI with Fresh Start reduces: (1) the average number of drinks per drinking day, (2) number of heavy episodic drinking days, and (3) number of alcohol-related problems in the past month, compared to BMI only, among college students mandated for alcohol intervention.

As a secondary aim, we will also test whether BMI with Fresh Start increases participants’ actual-ideal discrepancy, cognitive dissonance, or readiness to change.

## Methods: Patient and public involvement, trial design

### Patient and public involvement {11}

Patient perspectives and experiences have been centered throughout the development of Fresh Start. We used an iterative, user-centered design process that included 47 young adults who met the characteristics of the target population (hazardous drinking). Their feedback directly shaped key design features of the experience, such as the plot framing, narrative structure, game mechanics, and token selection. No formal patient or public advisory panel was convened during the design of this trial.

### Trial design {12}

This study is a randomized, longitudinal clinical trial. Participants will be randomized 1:1 to: (1) BMI plus VR (Fresh Start), or (2) a time- and content-matched BMI without any additional interventions (control). This design is intended to provide a preliminary test of the superiority of BMI with VR over BMI alone, and to generate the feasibility and tolerability data necessary to plan a larger, fully-powered effectiveness trial.

## Methods: Participants, interventions, and outcomes

### Trial setting {13}

The trial will be conducted at the University of Alabama’s (UA’s) Collegiate Recovery and Intervention Services (CRIS) center in Tuscaloosa, AL. CRIS regularly conducts screening, brief intervention, and weekly outpatient services with students referred for alcohol-related infractions, injuries, or arrests. Research staff at Brown University will create and maintain all data collection and participant management systems needed to conduct the research.

### Eligibility criteria {14a}

Participants will be (1) 18–34 years old, (2) report hazardous alcohol use in the past month (defined by NIAAA guidelines [72] as having had >5 drinks on a single occasion for men or >4 drinks for women, or >14 drinks in an average week for men, or >7 drinks per week for women), (3) speak and read English fluently, and (4) have been referred to UA’s CRIS for intervention after having received an alcohol-related infraction, injury, arrest, or other problem. Participants will be excluded if they (1) are at risk for or have a history of complicated alcohol withdrawal [73], (2) are currently receiving treatment for any substance use disorder, (3) screen positive for likely drug use disorder (DAST-10 > 3) [74, 75], (4) have severe mental illness (i.e., schizophrenia), or (5) have a history of significant motion sickness (Simulator Sickness Questionnaire [SSQ] score > 10) [76, 77] or other medical contraindications for using VR.

### Eligibility criteria for intervention deliverers {14b}

Study counselors will be master’s-level clinicians employed by or affiliated with UA’s CRIS center who are trained in the BASICS approach to brief motivational intervention. All counselors will complete a structured training protocol in the BMI procedure and in the use of Fresh Start before seeing participants and will be supervised throughout the trial by Dr. Barnett (see Trial monitoring, item {29}). This credential level is consistent with the clinical staff typically employed at CRIS and other college counseling centers to provide brief alcohol intervention.

### Intervention and comparator {15a}

**Figure 1:**
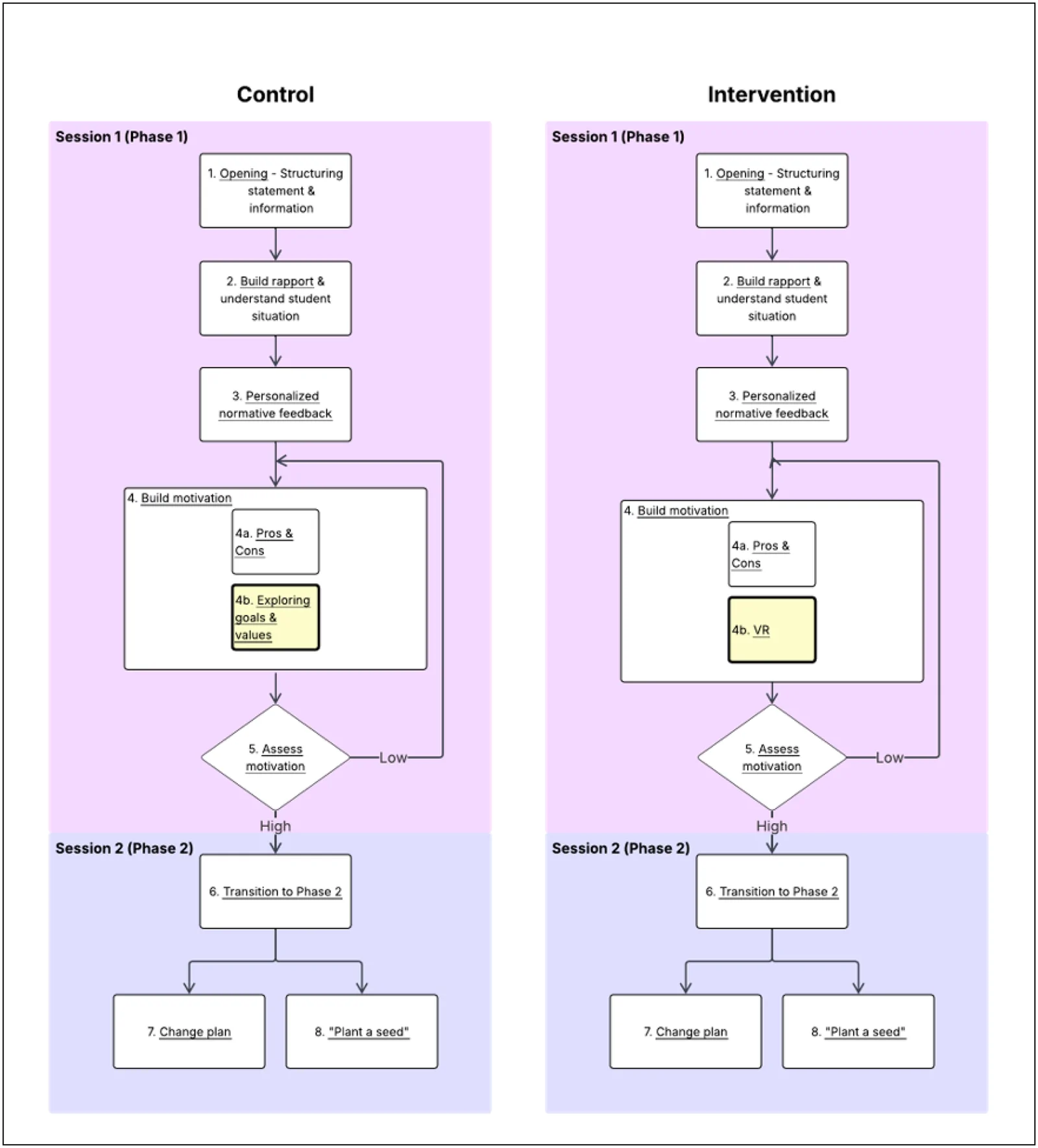
Flow of BMI sessions and topics by condition

Across both conditions, study counselors will deliver a BMI based on Barnett et al. (2019)[78]. This intervention is structured around two phases (see Figure 1). As shown in Figure 1, the control and intervention conditions differ only in step 4b of Phase 1, which is described in more detail below. Phase 1 opens with a structuring statement establishing the session’s collaborative, non-judgmental tone, followed by a review of the participant’s current drinking patterns. Counselors then present a personalized normative feedback report that compares participants’ drinking frequency, quantity, binge frequency, and estimated total monthly drinks against national norms for college students, displayed with percentile rankings. Counselors elicit participants’ reactions before guiding participants through a pros and cons discussion to elicit and reinforce change talk. Counselors then administer two motivational ruler items assessing importance and confidence around reducing drinking, using MI techniques throughout to elicit and reflect change talk. Phase 1 closes with a recapitulation summarizing key themes, including the participant’s own stated priorities, their reactions to the feedback about their drinking relative to peers, and any expressed ambivalence. Counselors will then summarize the session before concluding session 1.

Session 2 will generally take place at least a week after session 1. This session will start with an evaluation of motivation to change as in session 1 and will continue with questions designed to elicit recall from session 1. The counselors will be prepared to provide their recollection of the prior session and will probe for additional participant experiences or thoughts since that session. If the participant expresses interest in changing their drinking, counselors will complete Phase 2 of the BMI, which entails creating a change plan with goals for reducing drinking and strategies for accomplishing the goal. This phase includes discussion about challenges the participant anticipates and who among their friends or family might be supportive of their plan to change. For participants who do not express interest in changing their drinking, the session will continue as a “planting a seed” discussion, including open-ended questions about what would need to happen in order for them to consider change. In all sessions, the counselors will utilize MI-consistent techniques including reflective listening and supporting self-efficacy to change. Both sessions will last approximately 60 minutes.

#### BMI with Fresh Start (VR) condition.

After completing the pros and cons discussion, the counselor introduces Fresh Start as a tool that may help the participant reflect further on how their drinking fits with their priorities and invites them to try the experience. If the participant agrees, the counselor fits them with the VR head-mounted display and orients them to the experience. The user’s view is broadcast to a computer monitor so the counselor can observe in real time the participant’s actions and choices.

The Fresh Start experience begins by orienting users to VR and its controls, followed by a brief survey assessing the typical frequency and quantity of user’s alcohol use, and typical alcohol type (beer, wine, liquor/cocktails), which is used to customize the final scene. An introduction then provides context for the plot of the VR experience, which involves asking users to imagine that they are moving -- preparing to leave home to begin a new phase in life. Users are then free to explore the rooms of their virtual apartment, where they encounter a range of objects, or “tokens,” each representing a distinct life priority (e.g., a briefcase for career, wedding rings for romantic partnership, prayer beads for spirituality); as users select each token, a voice-over narrates what the object represents and why it might matter for the next chapter of their lives. Users can select as many tokens as they wish and adjust the relative size of each to reflect how much weight they give each priority. Once they have finished exploring the apartment, users are guided outside to a moving van, where they discover that a portion of the truck’s storage space is already occupied by a physical representation of alcohol, sized according to how much they reported drinking in the initial survey. Users must then decide which tokens to load into the remaining space and, if needed, whether to remove some of the alcohol to make room, physically enacting the trade-off between their drinking and the priorities they have chosen. Unless users choose very few priorities *and* have very low drinking, they must always choose to leave behind either some priorities or some alcohol, forcing users to actively resolve the conflict between alcohol use and their priorities. When users have finished loading the truck, a summary screen shows them what they elected to pack and what they chose to leave behind, inviting users to consider whether the choices they made truly reflect what matters most to them.

After the experience is complete (approximately 12–15 minutes), the counselor discusses it with the participant, using the participant’s specific token selections and choices in the van scene as a launching point for change talk. Counselors then ask the participant to consider how the experience prompted them to reflect on the role alcohol plays in their lives and uses MI techniques to elicit change talk based on their responses.

#### BMI alone (control).

In the BMI alone condition, counselors will engage participants in the “looking forward, looking back” exercise in place of the VR experience, with the goal of engaging in these discussions for a roughly equivalent amount of time as the VR (12–15 minutes).

### Explanation for choice of comparator {9b}

In the trial, we will compare BMI with VR (Fresh Start) versus BMI only. We made this decision because the BMI-only control condition represents the current standard of care for this population and setting and is matched on time and content to the other condition to isolate the effects of VR.

### Criteria for discontinuing or modifying allocated interventions {15b}

The interventions will not be modified for any reason during the trial. Participants who decline to use the VR when offered will continue to receive the BMI and will be retained in analyses in intent-to-treat fashion. Refusal and discontinuation rates will be tracked, analyzed, and reported, given that they are relevant to the acceptability, feasibility, and tolerability of VR (objective 1).

### Strategies to optimize adherence to interventions {15c}

Study clinicians will provide the assigned intervention at session 1 and then session 2 at least a week later, with follow-up phone calls, emails, and texts to encourage adherence if needed.

### Concomitant care permitted or prohibited during the trial {15d}

Participants are not required to abstain from any treatments or services during the trial. Those currently receiving treatment for a substance use disorder are excluded at enrollment, but participants may begin seeking such services during the trial period. Participation in any concurrent care will be tracked in follow-up surveys.

### Outcomes {16}

For objective 1, primary outcomes include tolerability, feasibility, and acceptability of VR used within a BMI. These outcomes will be assessed via post-intervention surveys in the following ways:

- Feasibility: proportion of BMI+VR participants who decline to use the program or stop prematurely, and their reasons for doing so. The Feasibility of Intervention Measure [79] will assess participants’ perceptions of whether using the VR in a BMI session is possible or doable.
- Tolerability: Simulator Sickness Questionnaire (SSQ) [77] assessing common symptoms of VR-related sickness (e.g., nausea, dizziness, headache, eye strain)
- Acceptability: Acceptability of Intervention Measure [79]
- Satisfaction: Client Satisfaction Questionnaire (CSQ-8) [80]

For objective 2, primary outcomes include alcohol use and related problems after intervention or control and will be assessed via online surveys at baseline, 1, 2, 3, and 6 months post-enrollment. Specific primary outcomes include:

- Average number of drinks consumed per drinking day (past 28 days; assessed via online Timeline Followback [TLFB][81])
- Number of heavy episodic drinking days (>5 standard drinks on a single occasion for men, >4 for women) in the past 28 days (TLFB)
- Number of alcohol-related problems in the past 28 days (Brief Young Adult Alcohol Consequences Questionnaire [BYAACQ] [82])

Exploratory analyses on potential mechanisms of intervention effects will be assessed via post-intervention and follow-up surveys and will include:

- Actual-ideal drinking discrepancy, assessed using a single-item scale on which participants rate how “far their current drinking patterns are from their personal ‘ideal,’ given who they are as a person” (0 [I’m not at my ideal] to 10 [I am extremely far from my ideal]) [22]
- Cognitive dissonance, assessed using the 24-item Dissonance Thermometer,[22] which assesses general discomfort resulting from a sense of discrepancy and negative self-referent emotions (e.g., guilt, regret)
- Readiness to change, as assessed by the Readiness to Change Questionnaire - Treatment Version [83]

### Harms {17}

There are no serious adverse events expected, as this is a minimal risk study. Potential VR-related harms include simulator sickness (nausea, dizziness, headache, or eye strain) and minor psychological discomfort from reflecting on one’s drinking. Both are assessed systematically in post-session survey instruments (SSQ and participant checklists) and follow-up surveys. Simulator sickness also is tracked actively during VR sessions, and is minimized through the exclusion of participants with a history of significant motion sickness. If an adverse event is reported, the contact PI (Dr. Wray) will complete an adverse events form and report the event to the Brown University IRB within 24 hours. A brief report of adverse events will be generated for the study record each year and forwarded to the IRB and sponsor. All adverse events will be reported in trial publications.

### Participant timeline {18}

**Figure 2:**
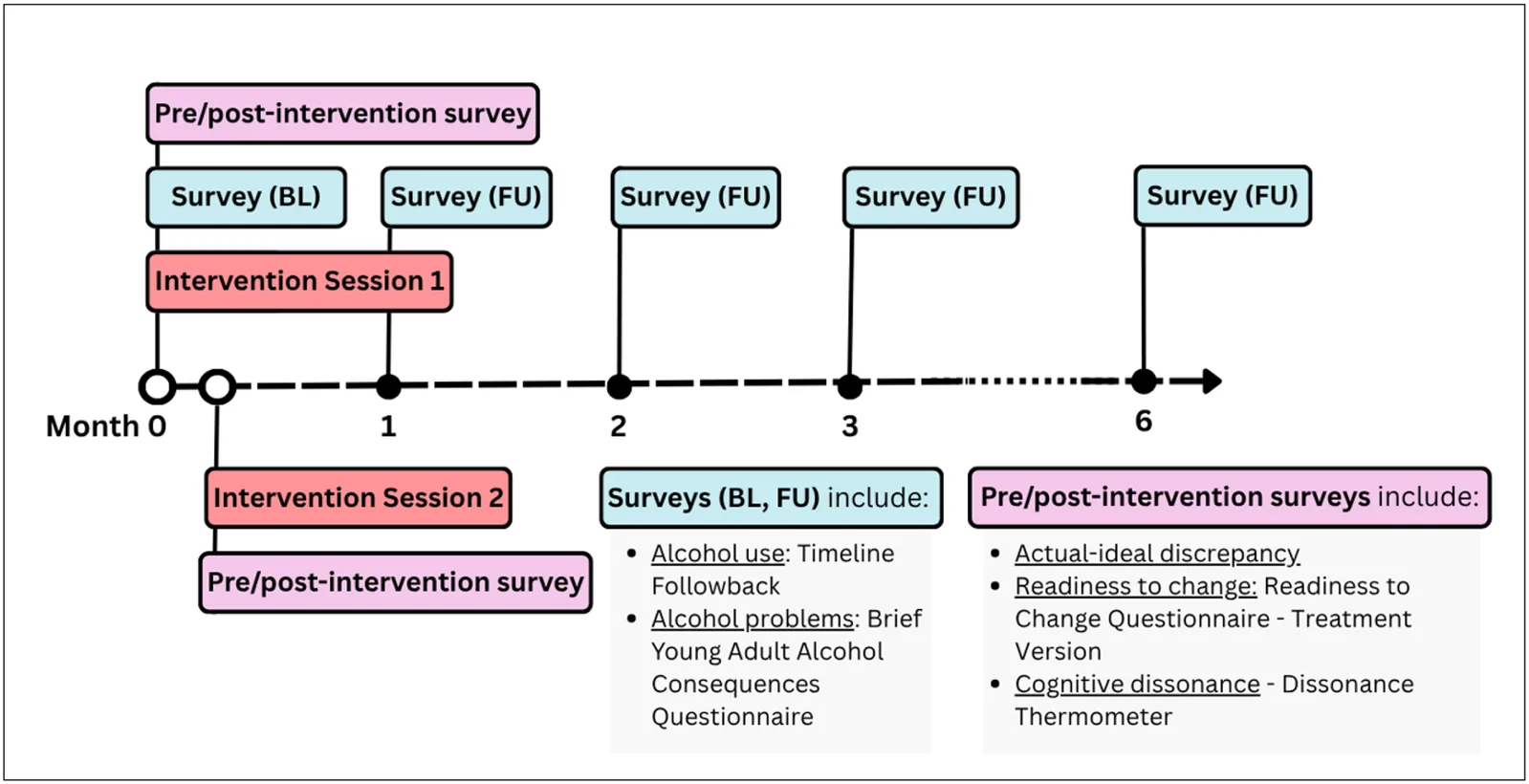
Participant timeline

Participants will be enrolled and randomized on a rolling basis beginning in October 2026, until the target sample of N=120 is reached. Once enrolled, participants will complete the assigned intervention in two sessions, separated by at least seven days. Participants will then complete online follow-up surveys at 1, 2, 3, and 6 months post-intervention. See Figure 2 for a depiction of the participant timeline.

### Sample size {19}

**Figure 3:**
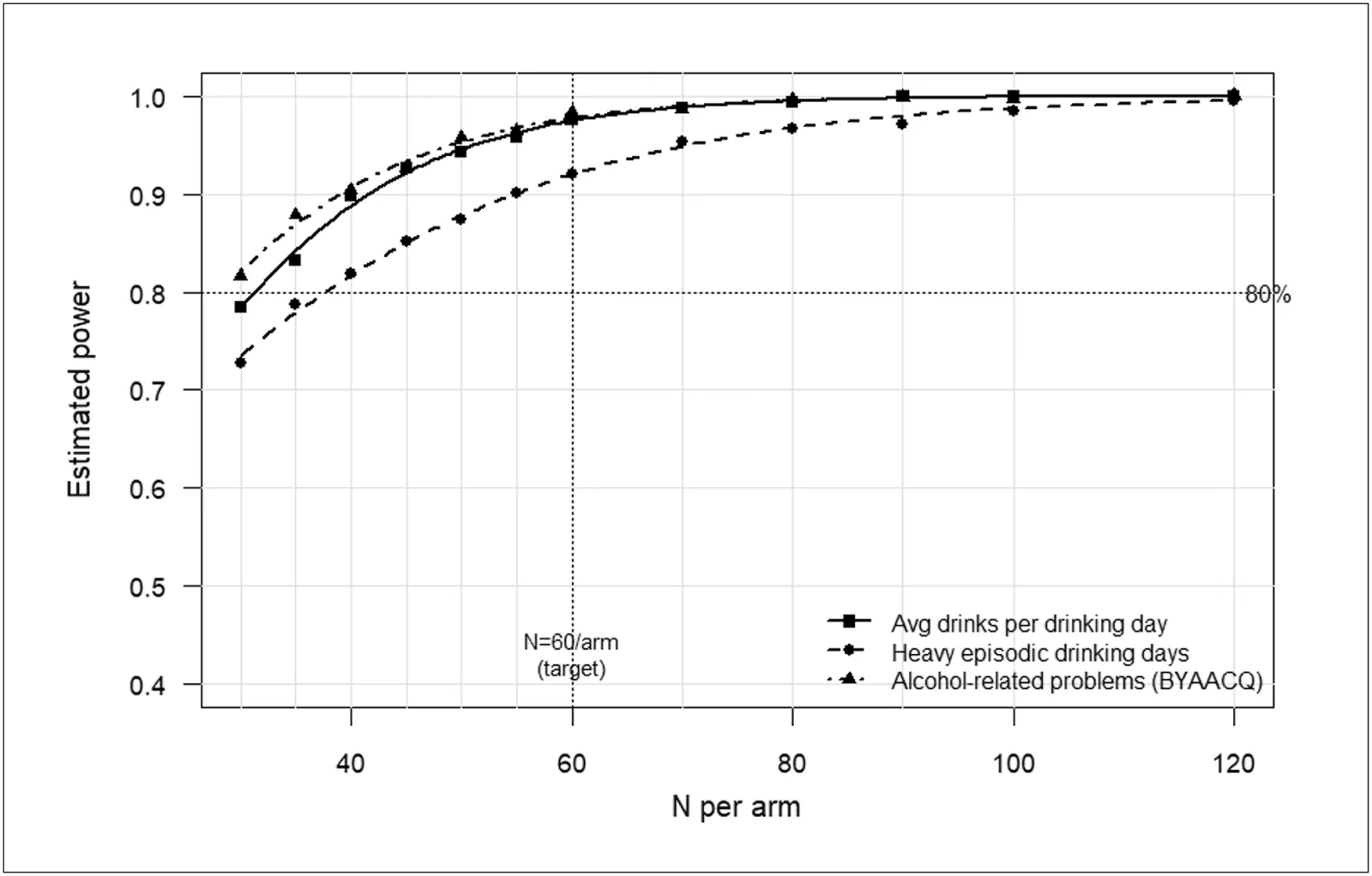
Power curves from Monte Carlo simulations by trial outcome

The proposed project is a pilot efficacy trial intended to provide a preliminary test of the VR experience’s efficacy in changing alcohol outcomes and to generate feasibility and tolerability data for a larger trial. Although pilot trials are not typically powered to detect statistically significant differences between conditions, establishing a reasonable sample size that provides meaningful preliminary evidence of efficacy can ensure that the trial is neither so small as to be uninformative nor larger than necessary for a developmental study. To that end, we conducted a simulation-based power analysis using the *MASS* package in R. We used 2,000 Monte Carlo replications per cell across a range of per-arm sample sizes (N = 30 to 120), assuming small-to-medium treatment effects that were consistent with those reported in past studies of BMIs with college students [28]. We specified uniform within-persons correlations (ρ=0.40) across all three outcomes. Aligning with trial design, we modeled four post-baseline assessment waves (months 1, 2, 3, and 6) with monotone attrition reaching approximately 15% by the final wave. These analyses indicated that N = 60 per arm (120 total) achieves power of 0.80 or greater across all three primary outcomes at alpha = 0.05, with heavy episodic drinking days as the outcome that required the largest sample size (see Figure 3). Our target sample of 120 is reasonable and relatively large for a pilot trial, providing a solid foundation for preliminary efficacy testing.

### Recruitment {20}

Participants will be recruited from among students referred to UA’s CRIS for alcohol intervention following receipt of alcohol-related infractions, injuries, arrests, or other incidents. At students’ initial visits to CRIS, staff will ask about their interest in participating in this study. If interested, they will be asked to complete a brief screening survey on a tablet or laptop computer. Approximately 60–80 students are referred to CRIS each year, suggesting it will be feasible to recruit 40 participants per year to reach N=120 across a three-year study timeline.

## Methods: Assignment of interventions

### Sequence generation {21a}

The allocation sequence will be determined using the computer-generated randomization module in Qualtrics. We will stratify each group by gender and those with scores >16 on the Alcohol Use Disorders Identification Test (AUDIT) [84] to ensure balance on these key variables.

### Type of randomization {21b}

Participants will be randomized using a simple 1:1 allocation without blocking. The allocation sequence will be generated before the study begins and will remain concealed from all personnel who enroll or interact with participants.

### Allocation concealment mechanism {22}

The Qualtrics platform will automatically assign participants to study conditions immediately upon completion of the baseline survey, without any interaction with study staff.

### Implementation {23}

The Qualtrics platform will generate the allocation sequence, enroll participants, and assign them to intervention conditions. Participants will be randomized 1:1 to: (1) BMI with VR (Fresh Start), or (2) a time- and content-matched BMI without any adjunctive interventions (control). Research staff who enroll participants will not have access to the allocation sequence prior to assignment.

### Blinding {24a}

Participants will not be blinded to study condition. However, research staff in charge of corresponding with participants will be unaware of participants’ condition assignment. The overall risk of bias is low because follow-up surveys are administered automatically by the study database.

### How blinding will be achieved {24b}

Due to the nature of the interventions, blinding of participants or study counselors delivering the interventions is not possible.

## Methods: Data collection, management, and analysis

### Data collection methods {25a}

We will use online surveys to collect all self-report data. Screening assessments will collect demographics, alcohol use patterns (TLFB [81, 85]), risk for alcohol use disorder (AUDIT [84]), risk for complicated alcohol withdrawal, drug-related problems (Drug Abuse Screening Test 10 [75]), risk for simulator sickness (SSQ [77]), and other eligibility criteria. Assessments for evaluating objective 1 outcomes (tolerability, acceptability, satisfaction) will be collected through an online survey administered immediately after each session. Assessments for evaluating objective 2 outcomes (average drinks per drinking day, number of heavy drinking days, alcohol-related problems) will be collected through online surveys at baseline, 1, 2, 3, and 6 months post-enrollment. On each follow-up survey’s due date, the study database will automatically send participants an email with instructions and a link to the survey. Participants will be asked to complete surveys within five days. After five days, research staff will follow up by phone and text. A post-session survey will assess tolerability, acceptability, satisfaction, and putative mechanism variables immediately following the initial session across all groups.

### Participant retention {25b}

For participants who do not complete follow-up surveys by the due date, reminder emails will be sent every other day for five days. If participants have not completed the survey within five days, research staff will contact them by phone, text, and email. Our previous studies using a similar approach yielded response rates >85% over similar 6-month follow-up periods.[86–88] Participants will be paid $50 for completing both intervention sessions and $30 for each monthly follow-up survey, with a bonus of $50 for completing all sessions and assessments ($190 total possible).

### Data management {26}

Data will be collected using Qualtrics and will be continuously synced with a database hosted on Brown servers. Prior to performing analyses, we will conduct range checks to ensure the plausibility of values. All participants will be assigned a unique study ID number so that no personal information is linked with research data. All study databases and survey datasets will be stored on Brown University’s internal servers, accessible only to essential research staff with two-step verification.

### Statistical methods {27a}

To address objective 1, we will calculate summary statistics and percentages of BMI with VR participants who declined to use the program or discontinued prematurely, as well as endorsement of specific symptoms/side effects (SSQ). We will calculate total scores and summary statistics for measures of acceptability and satisfaction and compare with the control group using *t*-tests.

To test the VR’s effects on primary drinking outcomes compared to the control conditions (objective 2), we will estimate several mixed models with dummy-coded treatment condition as a focal predictor and time (month) as a covariate. As most outcomes are count variables that tend to be over-dispersed, we will specify a Poisson or negative binomial distribution and use a log-link function. Incidence rate ratios (IRRs) and means across groups will be used to evaluate intervention effects.

For the exploratory aim, we will use multilevel mediation methods [89, 90] to test whether there is a significant indirect effect of BMI with VR on drinking outcomes changes in actual-ideal drinking discrepancy, cognitive dissonance, or readiness to change. This analysis will provide evidence regarding whether the intervention, related to control, affects the putative mediators in the expected direction and whether those mediators show associations with drinking outcomes.

### Analysis population {27b}

All analyses will be conducted in Intent-to-Treat (ITT) fashion,[91] meaning that participants who were randomized will be retained in analyses regardless of whether they completed the intervention. We will use full information maximum likelihood in estimating all models, which is robust to attrition.[92]

### Missing data {27c}

Data from those who drop out or withdraw will be used in analyses in ITT fashion.[91] We will use full information maximum likelihood (FIML) for missing data in all primary analyses.[92] Our procedures are designed to minimize missing data and attrition. We have been successful in achieving less than 15% missing data in similar studies.[86–88]

### Additional analyses {27d}

No formal subgroup or sensitivity analyses are pre-specified for primary outcomes. Exploratory mediation analyses are described under Statistical methods above. No other additional analyses are planned a priori.

## Methods: Monitoring

### Data monitoring committee {28a}

As this trial tests an intervention (BMI) known to improve alcohol-related outcomes, and the primary additional component (VR) is well-tolerated based on the existing literature, the risk associated with the interventions studied is insufficient to warrant close, independent monitoring of the study results beyond that already provided by the IRBs.

### Interim analyses and stopping guidelines {28b}

No interim analyses will be performed. The trial will be terminated when the target sample size is reached.

### Trial monitoring {29}

To evaluate the fidelity of BMI delivery across conditions and therapists, Dr. Magill will lead the coding of audiotaped sessions using the Motivational Interviewing Treatment Integrity (MITI) coding manual, version 4.2.1.[93] Twenty-minute segments of a randomly-selected 25% of session audio files will be double-coded by Brown University staff with extensive training in MI process research and intervention fidelity monitoring.[94–96] To assess interrater reliability, we will calculate intraclass correlation coefficients (two-way mixed, single measure absolute agreement) for behavioral count items and Cohen’s quadratic weight Kappa for global items. To evaluate BMI fidelity, we will compare MITI-rated summary indicators (question to reflection ratio, percent complex reflection, percent MI-adherent, and the Technical and Relational globals) across the two conditions.[93]

## Ethics

### Research ethics approval {30}

The proposed research is non-exempt human subjects research and will be conducted in accordance with 45 CFR Part 46. All procedures have been reviewed and approved by the Brown University Institutional Review Board (IRB; STUDY00001250).

### Protocol amendments {31}

Amendments to the trial protocol will first be submitted for review to the Brown University IRB, the IRB of record for this study. Once approved, these amendments will be reflected in the trial protocol, and a revised version will be updated on the trial registration page. Major changes to the scientific direction of the project will be requested from the study’s sponsor and approved prior to changing the conduct of the trial.

### Informed consent {32a}

Research staff will verbally review all key aspects of the study with participants, including the length of the study, what will be required of participants, principal risks, benefits, compensation, and confidentiality. Participants will also be explicitly informed that their research data will not be shared with the university. They will also be informed that they may withdraw from the research without penalty, but that, if they elect to do so and do not complete the two required counseling sessions they were mandated to complete, this may be shared with the university. The university could then require them to meet the conditions of their sanction in some other way. Throughout the consent process, staff will query participants periodically to ensure understanding. Once all participant questions have been answered and participants verbally affirm their consent to participate, consent will be documented on IRB-approved consent forms. Research staff will be trained to recognize cases when potential participants may not have the capacity to consent (e.g., those who may be under the influence of alcohol or drugs at enrollment), and these participants will not be enrolled.

### Additional consent provisions {32b}

Participants will be asked to provide consent for collection and broad use of their survey data to facilitate primary and secondary data analyses. Consistent with NIAAA policy, participants will also be asked if they are willing to opt-in to having their study data archived with the NIAAA Data Archive. Participants can opt-out of archiving and still participate without penalty.

### Confidentiality {33}

All members of the research staff will receive thorough training in procedures designed to maintain data security before beginning their work on the study. All digital audio and video collected will be reviewed by research staff within at least one month of their creation to remove any identifying information. De-identified files will be saved on Brown University’s secure servers, protected by two-step verification. Study surveys will be developed and hosted using Qualtrics, which is qualified to collect and store highly sensitive data, having achieved accreditations from FEDRAMP and HITRUST and earned ISO 27001 and SOC2 Type 2 certifications. Participants will be instructed to complete online surveys in a private location. Reminder emails will contain no identifying information or references to the study topic.

### Ancillary and post-trial care {34}

All participants will be provided with a list of referrals for alcohol/drug treatment programs and other mental health resources upon session completion. Participants who express distress or high-risk alcohol use at any point will be provided with crisis intervention, referrals, or contact information for emergency services, as appropriate. These events will be promptly reported to the Brown University IRB, which will serve as the IRB of record. There are no plans to provide compensation for trial-related harms beyond referral to appropriate clinical services.

## Discussion

This study will provide one of the first rigorous evaluations of a VR experience designed to help build motivation to change drinking. Across several reviews of VR experiences for addiction, [51–54] no experiences aimed at helping users resolve ambivalence about their drinking and talk about change have been developed or tested. This work represents a genuinely novel application of VR that helps patients reflect on how their drinking conflicts with their own values and priorities, with the goal of building intrinsic motivation to change. If this trial and others support the promise of this approach, our vision is that this tool (and other similar ones) could be used as part of any intervention that is compatible with the spirit and principles of MI.

This work is also unique in that, from the earliest stages of design and development, we devoted significant attention to ensuring that Fresh Start could realistically be implemented in real-world settings, such as counseling centers and addiction treatment facilities. Prior reviews have noted that many VR experiences developed for addiction treatment remain difficult to deploy in practice, [51, 54] due to barriers such as reliance on specialized infrastructure (e.g., hardware that requires headsets to be physically tethered to a computer or used only in rooms equipped with mounted base stations) or extensive training demands for patients and clinicians. By drawing on prior work exploring counselors’ perceived barriers to VR adoption and patients’ perspectives on its use [97–99] before making key design decisions, we were able to consider ways of addressing these barriers in the experience’s design. For example, because tethered hardware and fixed room requirements are likely to pose significant practical challenges in busy clinical settings, Fresh Start was built for the Meta Quest family of standalone, wireless head-mounted displays. The experience also includes a brief onboarding sequence that orients users to the hand controllers and movement mechanics, enabling even VR-naive users to acclimate within a few minutes. If experiences like Fresh Start prove beneficial, future work can build on this foundation by designing and evaluating explicit implementation strategies to support adoption across a range of treatment settings.

There is good reason to expect that a VR experience designed with implementation in mind could realistically be adopted in clinical settings. VR-assisted treatment for PTSD has been successfully deployed at dozens of VA hospitals and clinics, [100] counselor directories maintained by organizations such as XRHealth and VR Therapists International now list hundreds of providers offering VR-assisted services, [101, 102] and adoption of VR has been widespread in outpatient physical rehabilitation and specialty hospital settings for several years. [103–106] Notably, some addiction treatment facilities appear to be exploring VR adoption even ahead of a definitive evidence base, suggesting that clinical enthusiasm for the technology is already outpacing the research. [107]

If the results of the proposed trial support the efficacy of VR-enhanced BMI, future work should examine its real-world effectiveness, test implementation strategies for clinic adoption, and explore pathways for commercialization. The pilot data that will be produced in this trial offer a strong foundation for this work.

## Figures and Tables

**TABLE 1.** Effect size assumptions by outcome

Outcome	*IRR*	Interpretation
Average drinks per drinking day	0.75	1 fewer drink
Heavy episodic drinking days	0.60	1.2 fewer days
Alcohol-related problems	0.75	2 fewer problems

## Data Availability

The datasets generated and/or analyzed during the current study will be made available after study completion. A de-identified archival dataset will be uploaded to the NIMH Data Archive and made available to requesting investigators after an embargo period of 2 years.

## Strengths and limitations of this study:

- Strength: Rigorous randomized-controlled design with a meaningful follow-up period
- Strength: Explores whether integrating VR within an existing, evidence-based counseling approach confers benefit
- Strength: Tests preliminary efficacy and feasibility in an especially relevant population: College students who have been mandated receive counseling due to their drinking
- Weakness: Trial will be conducted at a single site (the University of Alabama), and thus, generalizability to other populations and locations may be limited
- Weakness: Study is a pilot study, so it will not be large enough to be certain that any between-group effects can be detected if they are present

## Abbreviations

AUDIT: Alcohol Use Disorders Identification Test
BMI: Brief motivational intervention
BYAACQ: Brief Young Adult Alcohol Consequences Questionnaire
CIWA-Ar: Clinical Institute Withdrawal Assessment for Alcohol, Revised
CRIS: Collegiate Recovery and Intervention Services
CSQ-8: Client Satisfaction Questionnaire
DAST-10: Drug Abuse Screening Test 10
HMD: Head-mounted display
IRR: Incidence rate ratio
ITT: Intent-to-treat
MI: Motivational interviewing
MITI: Motivational Interviewing Treatment Integrity
RCT: Randomized controlled trial
SSQ: Simulator Sickness Questionnaire
TLFB: Timeline Followback
UA: University of Alabama
VR: Virtual reality
